# Appendiceal Intussusception Secondary to Endometriosis: A Rare Etiology of Right Lower Quadrant Abdominal Pain

**DOI:** 10.5334/jbsr.2739

**Published:** 2022-05-04

**Authors:** Cedric Trefois, Emmanuel Coche

**Affiliations:** 1 Cliniques universitaires Saint-Luc, BE

**Keywords:** appendiceal intussusception, invagination, appendix, endometriosis, ultrasonography, computed tomography

## Abstract

**Teaching Point:** Appendiceal intussusception appears as a “sausage or target shaped” lesion in the caecal lumen and may be caused by a “lead point”.

## Case History

**Figure 1:**
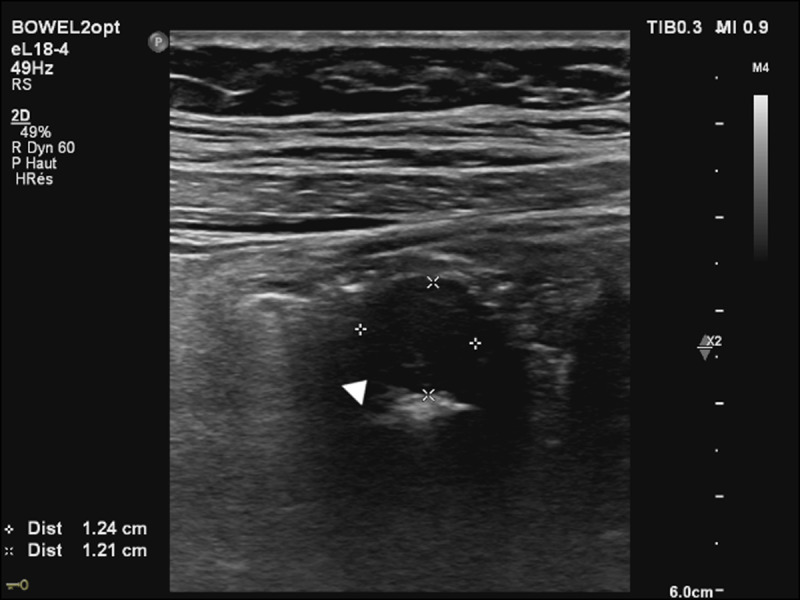


**Figure 2:**
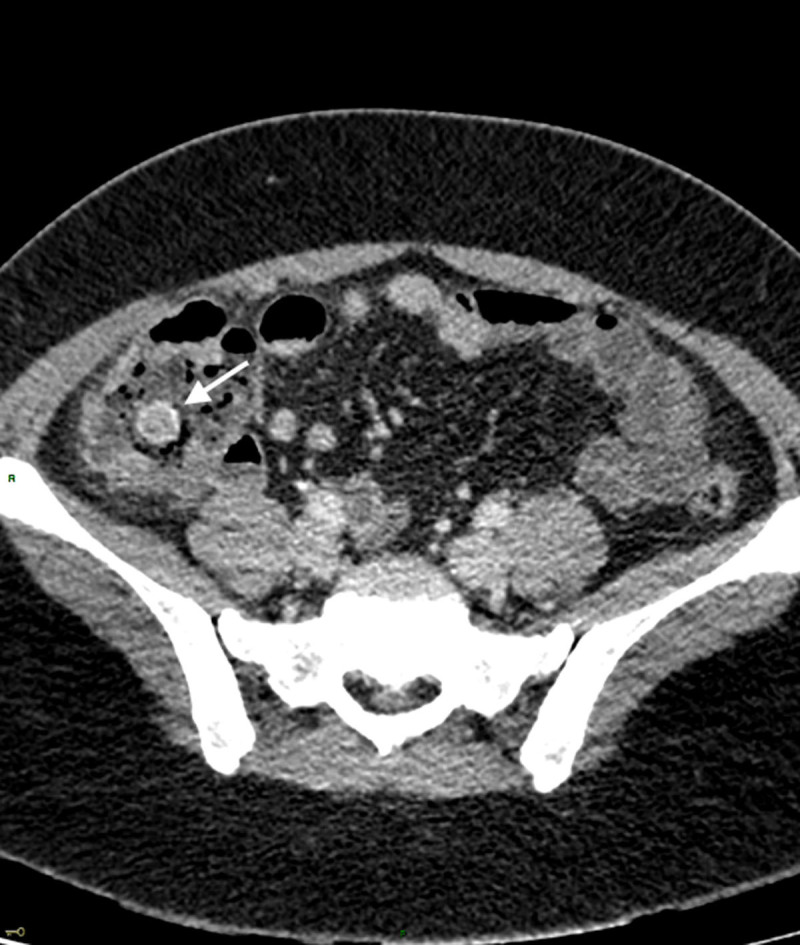


**Figure 3:**
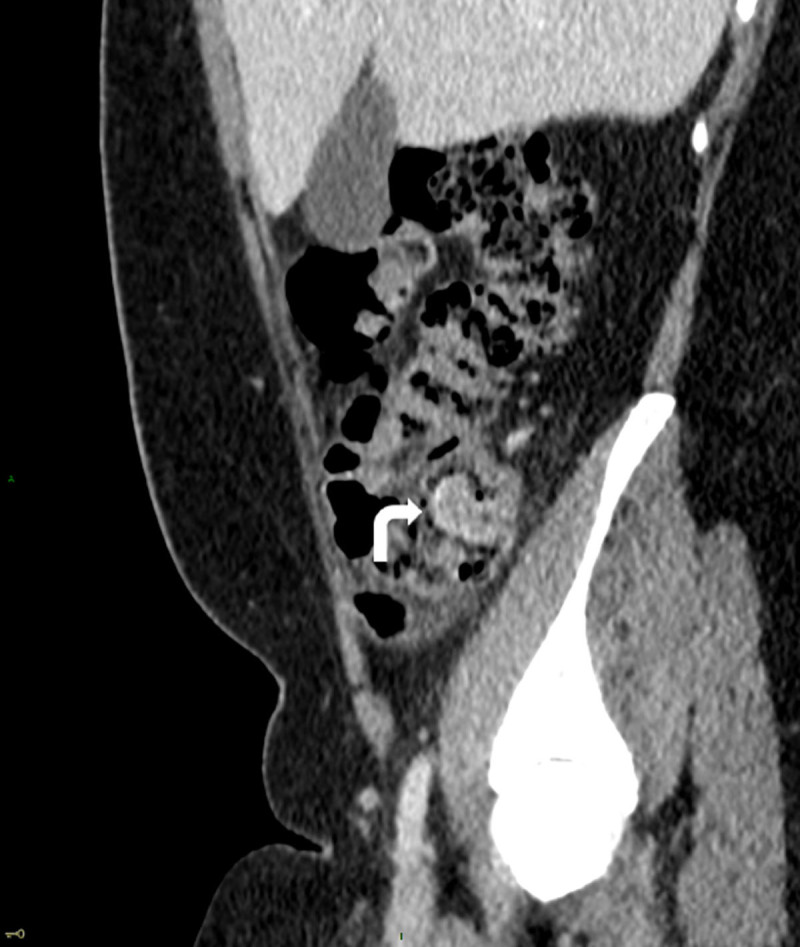


A 30-year-old woman was admitted to the emergency department for a 24-hour history of abdominal pain. She denied fever and did not report any other symptoms. Clinical examination revealed abdominal tenderness in the right lower quadrant. Laboratory tests demonstrated a CRP at the upper limit of the normal range (5mg/L). The patient was known to have endometriosis with a previous history of ovarian endometrioma. An abdominal ultrasound revealed a tubular lesion with concentric layers in the caecal lumen (***Figure 1***, arrowhead). A contrast-enhanced computed tomography (CT) of the abdomen was then carried out, revealing a lesion in the caecal lumen demonstrating mucosal enhancement continuous with the caecal mucosa. The lesion appeared axially as a target (***Figure 2***, arrow) and sagittally as a sausage (***Figure 3***, curved arrow), compatible with an appendiceal intussusception without evidence for an associated appendicitis. A complementary colonoscopy was performed, confirming this diagnosis. Given the favorable clinical course, conservative treatment was instituted followed by a partial caecectomy one month later. Pathology confirmed an invaginated appendix demonstrating endometriosis implants at its base.

## Comment

Appendiceal intussusception is a rare condition, accounting for around 0.01% of appendectomies. The pathology affects predominantly adults, in particular women who in 70% of cases present a favorable underlying pathology, notably endometriosis, appendiceal mucocele, adenoma, adenocarcinoma, and Crohn’s disease. Pediatric patients, on the other hand, more frequently present with an underlying local inflammatory pathology. The condition is more often than not asymptomatic but can present as relatively isolated pain in the right lower quadrant. The pathology is often diagnosed incidentally during colonoscopy; however, radiologic imaging diagnosis is obtained more frequently with CT given the low sensitivity of ultrasound in digestive imaging. In the latter case, the invaginated appendix appears as a target or sausage shape within the caecal lumen demonstrating continuous mucosal enhancement with the caecal mucosa. Treatment depends on the presence or lack of an underlying pathology: colonoscopy or partial caecotomy with appendectomy in the absence of a malignant lesion and right hemicolectomy in the presence of a malignant lesion [1]. In conclusion, this is a rare pathology, often asymptomatic, and its imaging diagnosis should raise the suspicion of an underlying benign or malignant etiology.
